# Supplementary material for: Anionic amino acids support hydrolysis of poly-β-(1,6)-N-acetylglucosamine exopolysaccharides by the biofilm dispersing glycosidase Dispersin B
Source: J Biol Chem. 2020 Dec 23;296:100203. doi: 10.1074/jbc.RA120.015524 (PMC7949127; doi:10.1074/jbc.RA120.015524)
Supplement: Figures S1–S8 [file mmc1.pdf]

Supporting Information:

Anionic amino acids support hydrolysis of poly-β-(1,6)-*N*-acetylglucosamine exopolysaccharides by the biofilm dispersing glycosidase Dispersin B

Alexandra P. Breslawec, Shaochi Wang, Crystal Li, and Myles B. Poulin*

Department of Chemistry and Biochemistry, University of Maryland, College Park, Maryland 20742, United States

*Corresponding Author: Myles B. Poulin

E-mail: mpoulin@umd.edu

Supporting Information

1. Experimental procedures S2

1.1. General S2

1.2. Preparation of trisaccharide analogs **1–4** S2

2. Supporting Information Figures S1–S8 S6

3. Spectral Data S14

1. EXPERIMENTAL PROCEDURES

1.1. General

All chemical reactions were carried out in oven-dried glassware under anhydrous conditions with freshly distilled solvents under a positive pressure of argon gas, and all chemicals purchased were reagent grade and used without further purification, unless otherwise noted. Reactions were monitored by thin-layer chromatography (TLC). Spots were visualized by UV light (254 nm) and charring with a solution of 10% sulfuric acid in MeOH. Column chromatography was performed on silica gel (200–300 mesh). ^{1}H and ^{13}C NMR spectra were recorded at room temperature for solutions in $CDCl_3$ or D_2O on Advance III-400 or III-600 spectrometers (Bruker). ^{1}H NMR and ^{13}C NMR chemical shifts are reported relative to residual solvent peak in parts per million (ppm). The following standard abbreviations are used to indicate multiplicity: s = singlet, d = doublet, t = triplet, m = multiplet, dd = doublet of doublets, dt = doublet of triplets, td = triplet of doublets and br = broad. ESI-MS experiments were performed on an AccuTOF-ESI mass spectrometer. MALDI-TOF mass spectra were performed on a Bruker Autoflex Speed spectrometer using trihydroxyacetophenone as the matrix, unless otherwise noted.

1.2. Synthesis of Trisaccharide Substrates

Trisaccharides **1** and **2**, and building blocks **4** and **5**, were prepared as described previously (33). Building block **6** was prepared from D-Glucose following literature procedures (59).

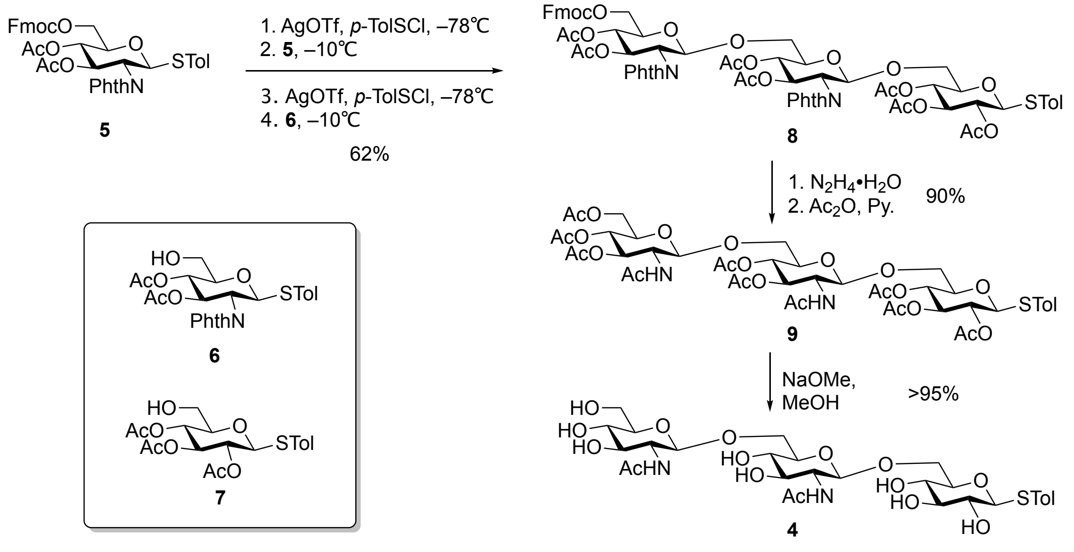

Scheme S1. Synthesis of trisaccharide 4.

***p*-Tolyl 3,4-di-*O*-acetyl-6-*O*-fluorenylmethyloxycarbonyl-2-deoxy-2-phthalimido-*β*-D-glucopyranosyl-(1→6)-3,4-di-*O*-acetyl-2-deoxy-2-phthalimido-*β*-D-glucopyranosyl-(1→6)-2,3,4-tri-*O*-acetyl-1-thio-*β*-D-glucopyranoside (8)**

Compound **8** was synthesized using a modification of the iterative one-pot glycosylation method reported previously (33). Briefly, a solution of glycosyl donor **5** (30 mg, 0.04 mmol) and freshly flame-activated 4 Å molecular sieves (150 mg) in CH_2Cl_2 (2 mL) was stirred at room temperature for 30 minutes, then cooled to –78°C. A solution of AgOTf (39 mg, 0.15 mmol) dissolved in Et_2O (1 mL) was added dropwise to the solution. After 5 min at –78°C, freshly distilled, orange colored *p*-TolSCl (7.9 μL, 0.060 mmol) was added using a micro-syringe. After the donor was consumed (as determined from TLC analysis, approximately 2~3 min at –78°C), a solution of acceptor **6** (21 mg, 0.04 mmol) in CH_2Cl_2 (0.2 mL) was introduced dropwise along the flask wall via a syringe to avoid heating the reaction mixture. The reaction mixture was then warmed to –10 °C over 20 min while stirring. After consumption of the acceptor was observed (TLC), the reaction mixture was cooled to –78°C, followed by the addition of AgOTf (39 mg, 0.15 mmol) in Et_2O (1 mL). After 5 min, additional *p*-TolSCl (6.3 μL, 0.048 mmol) was added using a microsyringe. When the disaccharide donor was completely consumed (~ 5 min at –78°C), a solution of acceptor **7** (19 mg, 0.038 mmol) in CH_2Cl_2 (0.2 mL) was added dropwise along the flask wall via a syringe. The reaction mixture was warmed to –10°C and stirred for an additional 30 min. The final reaction mixture was diluted with CH_2Cl_2 (20 mL) and filtered through a pad of celite. The CH_2Cl_2 phase was washed successively with a saturated aqueous solution of $NaHCO_3$ (2 × 20 mL) followed by brine (2 × 10 mL). The organic layer was dried with $MgSO_4$, concentrated, and purified by silica gel flash chromatography (EtOAc:hex = 2:3), to give 36 mg of **7** as a white amorphous solid (62% yield). ^{1}H NMR (600 MHz, $CDCl_3$) δ 7.91 – 7.86 (m, 2H), 7.79 (d, *J* = 7.5 Hz, 2H), 7.76 – 7.69 (m, 4H), 7.67 – 7.64 (m, 2H)7.63 – 7.58 (m, 2H), 7.42 (t, *J* = 7.4 Hz, 2H), 7.35 (tdd, *J* = 7.4, 4.2, 1.1 Hz, 2H), 7.18 (d, *J* = 8.0 Hz, 2H), 7.11 (d, *J* = 8.0 Hz, 2H), 5.79 (dd, *J* = 10.7, 9.0 Hz, 1H), 5.56 (dd, *J* = 10.7, 9.1 Hz, 1H), 5.51 (d, *J* = 8.5 Hz, 1H, H-1), 5.30 (d, *J* = 8.5 Hz, 1H, H-1'), 5.17 (dd, *J* = 10.1, 9.1 Hz, 1H), 5.05 (dd, *J* = 9.4, 9.3 Hz, 1H), 4.91 (dd, *J* = 10.1, 9.2 Hz, 1H), 4.71 (dd, J = 9.4, 9.3 Hz, 1H), 4.66 (dd, *J* = 9.8, 9.7 Hz, 1H), 4.44 (dd, *J* = 7.4, 3.8 Hz, 1H), 4.41 (d, *J* = 10.0 Hz, H-1''), 4.40 (dd, *J* = 5.5, 5.2 Hz), 4.37 (dd, *J* = 10.7, 8.5 Hz, 1H), 4.33 – 4.28 (m, 2H), 4.18 (dd, *J* = 10.7, 8.5 Hz, 1H), 3.97 – 3.91 (m, 1H), 3.85 (dd, *J* = 11.3, 2.4 Hz, 1H), 3.83 (dd, *J* = 10.9, 2.5 Hz, 1H), 3.67 (dd, *J* = 11.3, 5.9 Hz, 1H), 3.61 (ddd, *J* = 10.1, 5.9, 2.5 Hz, 1H), 3.48 (ddd, *J* =

9.8, 6.8, 2.5 Hz, 1H), 3.37 (dd, *J* = 10.9, 6.8 Hz, 1H), 2.36 (s, 3H), 2.02 (s, 6H), 1.90 (s, 3H), 1.90 (s, 3H), 1.88 (s, 3H), 1.86 (s, 3H), 1.77 (s, 3H). ^{13}C NMR (150 MHz, $CDCl_3$) δ 169.59, 169.54, 169.10, 168.93, 168.75, 168.65, 154.44, 142.93, 142.87, 140.83, 140.79, 137.92, 133.80, 133.47, 132.83, 129.23, 127.42, 127.40, 127.06, 126.75, 126.72, 124.83, 124.77, 123.21, 123.03, 119.57, 97.54, 97.31, 84.64, 76.18, 73.55, 72.68, 71.34, 70.30, 70.17, 69.72, 69.28, 68.86, 68.35, 68.20, 67.11, 65.47, 53.91, 46.20, 20.70, 20.23, 20.13, 20.06, 19.99, 19.94, 19.89. MALDI-TOF: 1407.11 $[M+Na]^+$, 1423.10 $[M+K]^+$.

***p*-Tolyl 3,4,6-tri-*O*-acetyl-2-acetamido-2-deoxy-*β*-D-glucopyranosyl-(1→6)-3,4-di-*O*-acetyl-2-acetamido-2-deoxy-*β*-D-glucopyranosyl-(1→6)-2,3,4-tri-*O*-acetyl-1-thio-*β*-D-glucopyranoside (9)**

Compound **8** (40 mg, 0.028 mmol) was treated with hydrazine monohydrate (10 mg, 0.20 mmol) in ethanol (3 mL). After stirring for 2 hours at reflux the mixture was concentrated under vacuum. The resulting residue was dissolved in pyridine (3 mL) to which acetic anhydride (1.5 mL, 15.8 mmol) was added. The mixture was stirred at room temperature for 16 h and diluted with CH_2Cl_2 and washed with water. The organic phase was dried over $MgSO_4$ and concentrated under vacuum. The crude product was purified by silica column chromatography (CH_2Cl_2:MeOH = 40:1) to give the per-acetylated trisaccharide **9** (26 mg, 90% yield). ^{1}H NMR (500 MHz, $CDCl_3$) δ 7.37 (d, *J* = 7.8 Hz, 2H), 7.18 (d, *J* = 7.8 Hz, 2H), 6.02 (d, *J* = 8.8 Hz, 1H, NH), 5.57 (d, *J* = 8.8 Hz, 1H, NH), 5.24 – 5.14 (m, 3H), 5.05 (dd, *J* = 9.6, 9.5 Hz, 1H), 4.99 (dd, *J* = 9.6, 9.5 Hz, 1H), 4.94 (dd, *J* = 9.8, 9.7 Hz, 1H), 4.86 (dd, *J* = 9.6, 9.5 Hz, 1H), 4.65 (d, *J* = 10.0 Hz, 1H, H-1), 4.52 (d, *J* = 8.4 Hz, 2H, H-1’, H-1’’), 4.26 (dd, *J* = 12.4, 4.6 Hz, 1H), 4.15 – 4.06 (m, 1H), 4.01 – 3.91 (m, 4H), 3.68 – 3.62 (m, 2H), 3.62 – 3.58 (m, 1H), 3.51 (dd, *J* = 11.6, 5.6 Hz, 1H), 3.46 (dd, *J* = 11.4, 5.4 Hz, 1H), 2.36 (s, 3H), 2.08 (s, 3H), 2.07 (s, 3H), 2.06 (s, 3H), 2.05 (s, 3H), 2.03 (s, 3H), 2.02 (s, 3H), 2.01 (s, 3H), 1.97 (s, 3H), 1.96 (s, 3H), 1.84 (s, 3H). ^{13}C NMR (125 MHz, $CDCl_3$) δ 170.80, 170.67 (2C), 170.36, 170.25, 170.19, 170.07, 169.93, 169.35, 169.22, 138.92, 133.46 (2C), 130.00 (2C), 127.65, 101.45, 101.10, 85.81, 73.92, 73.15, 73.00, 72.75, 72.01, 69.96, 68.85, 68.61, 68.58, 67.92, 67.59, 62.02, 54.23, 54.09, 23.22, 23.08, 21.14, 20.77, 20.74 (3C), 20.69, 20.66, 20.61, 20.56. ESI-TOF: 1029.4892 $[M+H]^+$, 1046.5235 $[M+NH_4]^+$.

***p*-Methylphenyl 2-acetamido-2-deoxy-*β*-D-glucopyranosyl-(1→6)-2-acetamido-2-deoxy-*β*-D-glucopyranosyl-(1→6)-1-thio-*β*-D-glucopyranoside (4)**

Compound **7** (20 mg, 0.019 mmol) was treated with 0.05 M NaOMe in MeOH (3 mL) at pH = 9. After stirring for 3 h at room temperature, the reaction mixture was neutralized by addition of Amberlite IR-120 (H+) resin. The mixture was filtered and evaporated under vacuum to give compound **3** (13 mg, quant.) as an amorphous white solid. ^{1}H NMR (600 MHz, D_2O) δ 7.36 (d, J = 7.7 Hz, 2H), 7.17 (d, J = 7.7 Hz, 2H), 4.63 (d, J = 9.9 Hz, 1H, H-1), 4.43 (d, J = 8.4 Hz, 1H, H-1'), 4.38 (d, J = 8.5 Hz, 1H, H-1''), 4.07 (d, J = 11.1 Hz, 1H), 3.98 (d, J = 11.5 Hz, 1H), 3.81 (d, J = 12.3 Hz, 1H), 3.66 – 3.57 (m, 5H), 3.48 – 3.45 (m, 2H), 3.42 – 3.36 (m, 3H), 3.34 – 3.33 (m, 2H), 3.32 – 3.23 (m, 2H), 3.20 (dd, J = 9.9, 9.4 Hz, 1H, H-2), 2.24 (s, 3H), 1.87 (s, 3H), 1.86 (s, 3H). ^{13}C NMR (150 MHz, D_2O) δ 173.99, 173.97, 138.40, 131.67 (2C), 129.61 (2C), 127.70, 100.95, 87.25, 78.19, 76.71, 75.36, 74.13, 73.38, 73.25, 71.13, 69.51, 69.41, 68.82, 68.02, 67.93, 60.22, 54.98, 54.92, 21.73, 21.68, 19.69. MALDI-TOF: 715.0405 $[M+Na]^+$.

2. SUPPORTING INFORMATION FIGURES

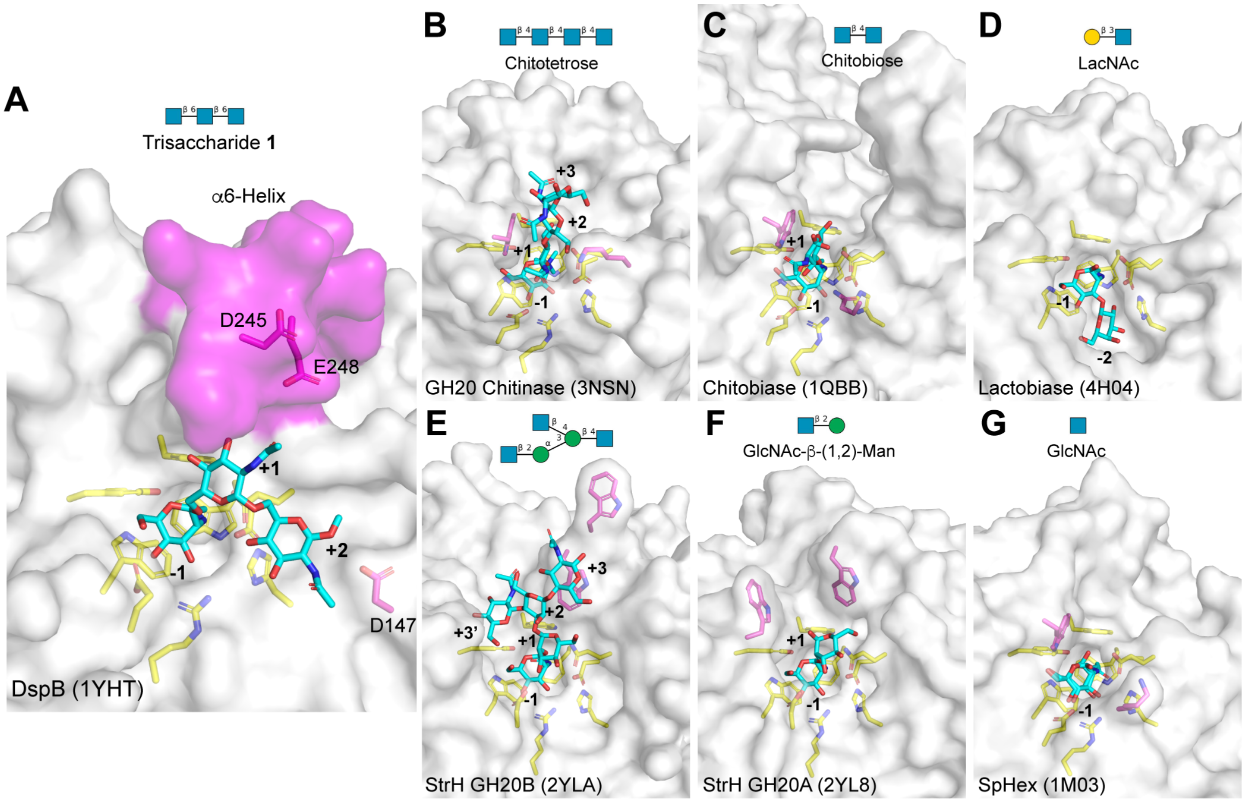

Figure S1. Substrate binding surface of DspB compared to GH20 orthologues. Structures are shown in surface representation with conserved active site amino acids involved in recognition of GlcNAc in the –1 binding site shown as yellow sticks. (A) Apo structure of DspB (PDB 1YHT) docked with trisaccharide analog **1**. The position of a α6-helix extension unique in the DspB structure is shown as a magenta surface. The position of anionic amino acids D147, D245, and E248 are shown as magenta sticks. (B) Structure of the GH20 domain of *Ostrinia furnacalis* Hex1 chitinase enzyme bound to chitotetrose (PDB 3NSN). (C) Structure of *Serratia marcescens* chitobiase GH20 domain bound to chitobiose (PDB 1QBB). (D) Structure of *Bifidobacterium bifidum* lacto-*N*-biase GH20 domain bound to LacNAc disaccharide (PDB 4H04). (E) Structure of the *Streptococcus pneumoniae* StrH enzyme GH20B domain bound to a bisected *N*-glycan oligosacharide (PDB 2YLA). (F) Structure of the *S. pneumoniae* GH20A domain bound to GlcNAc-β-(1,2)-Man disaccharide (PDB 2YL8). (G) Structure of *Streptomyces plicatus* β-*N*-hexosaminidase (SpHex) GH20 domain bound to GlcNAc (PDB 1M03). For (B)–(G) aromatic and cationic amino acids that are involved in recognition of the poly/oligosaccharide substrates are shown as magenta sticks.

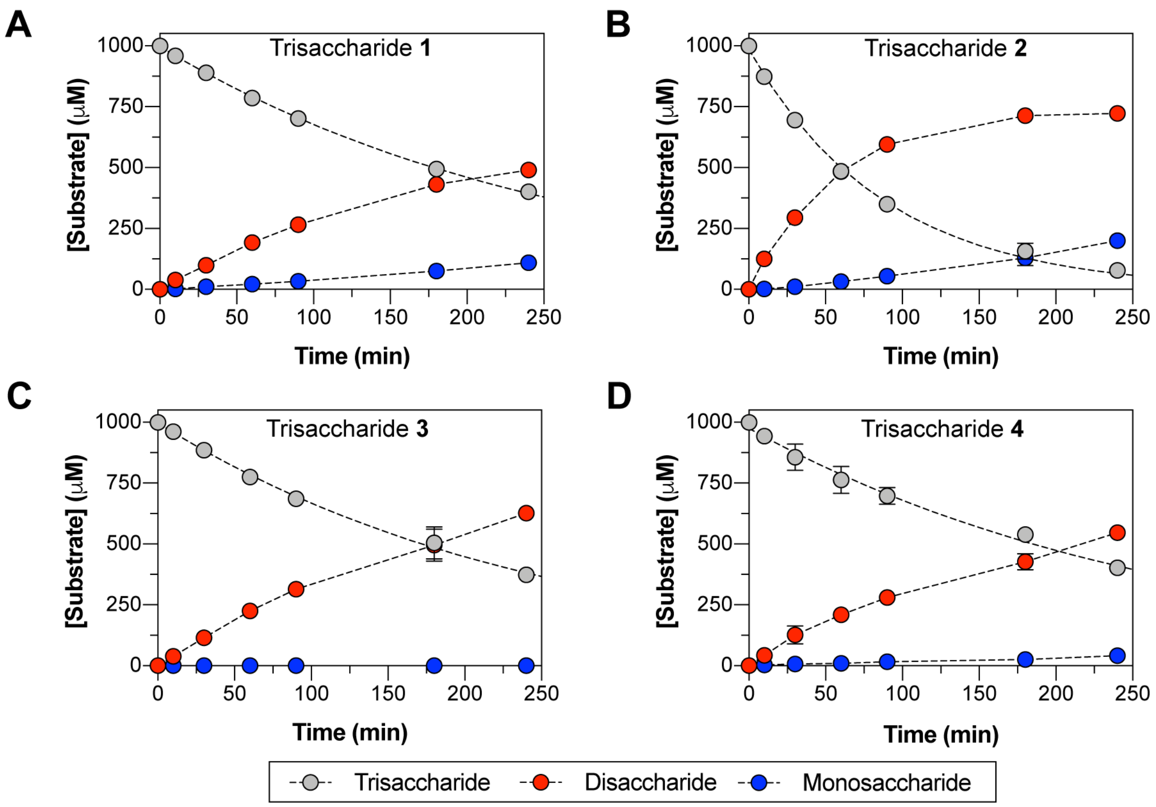

Figure S2. Hydrolysis of PNAG analogs 1–4 by DspB$_{wt}$. Reaction progress curves for hydrolysis reactions containing 18 μM DspB and 1000 μM of trisaccharide analog **1** (A), **2** (B), **3** (C) or **4** (D) measured as a function of time. Error bars represent the standard deviation from at least two replicate experiments. Lines were added to aid identification of the disappearance of the trisaccharide (grey) and appearance of reducing-end disaccharide (red) and reducing-end monosaccharide (blue) products.

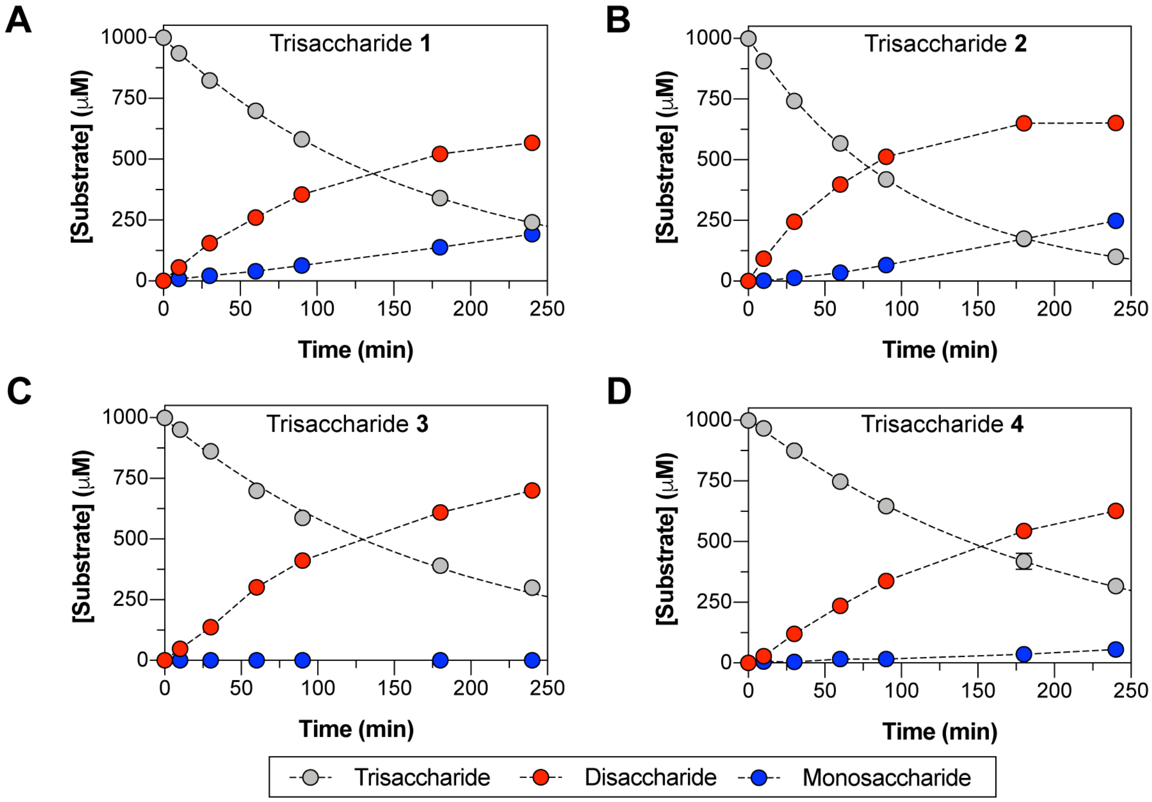

Figure S3. Hydrolysis of PNAG analogs 1–4 by DspB D147N mutant. Reaction progress curves for hydrolysis reactions containing 45 μM DspB D147N and 1000 μM of trisaccharide analog **1** (A), **2** (B), **3** (C) or **4** (D) measured as a function of time. Error bars represent the standard deviation from at least two replicate experiments. Lines were added to aid identification of the disappearance of the trisaccharide (grey) and appearance of reducing-end disaccharide (red) and reducing-end monosaccharide (blue) products.

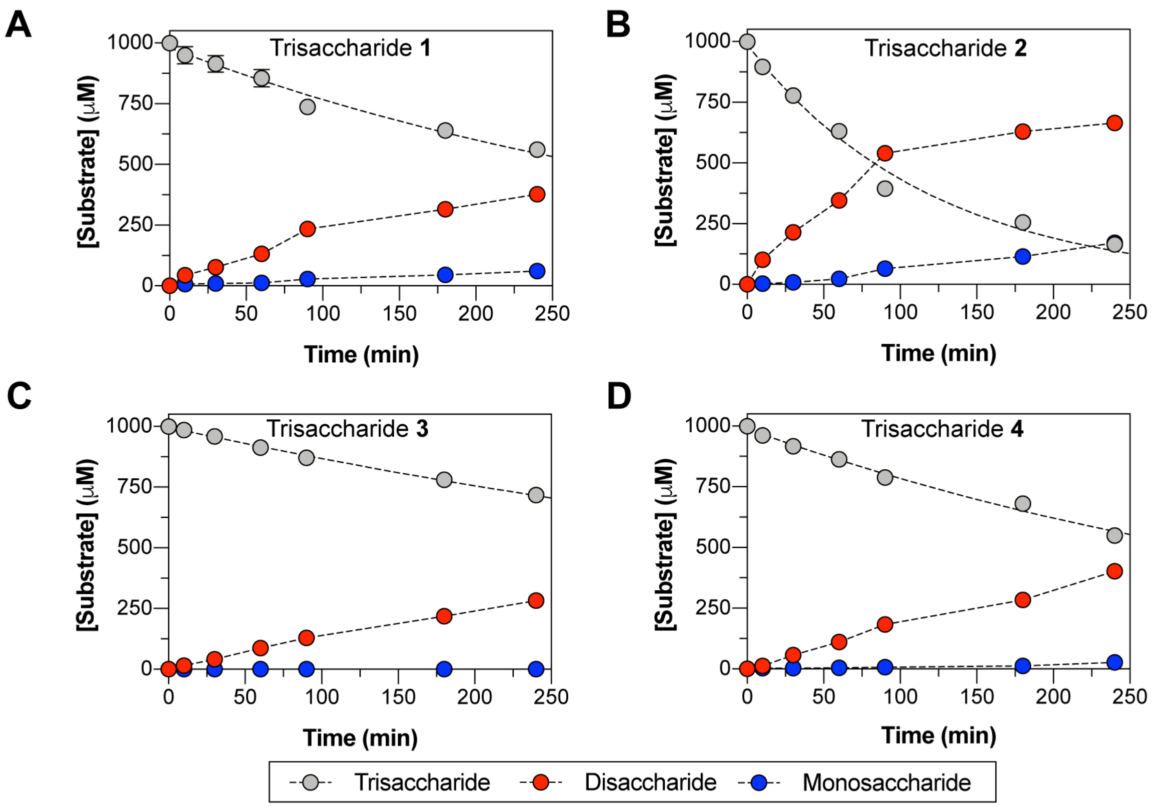

Figure S4. Hydrolysis of PNAG analogs 1–4 by DspB D245N mutant. Reaction progress curves for hydrolysis reactions containing 20 μM DspB D245N and 1000 μM of trisaccharide analog **1** (A), **2** (B), **3** (C) or **4** (D) measured as a function of time. Error bars represent the standard deviation from at least two replicate experiments. Lines were added to aid identification of the disappearance of the trisaccharide (grey) and appearance of reducing-end disaccharide (red) and reducing-end monosaccharide (blue) products.

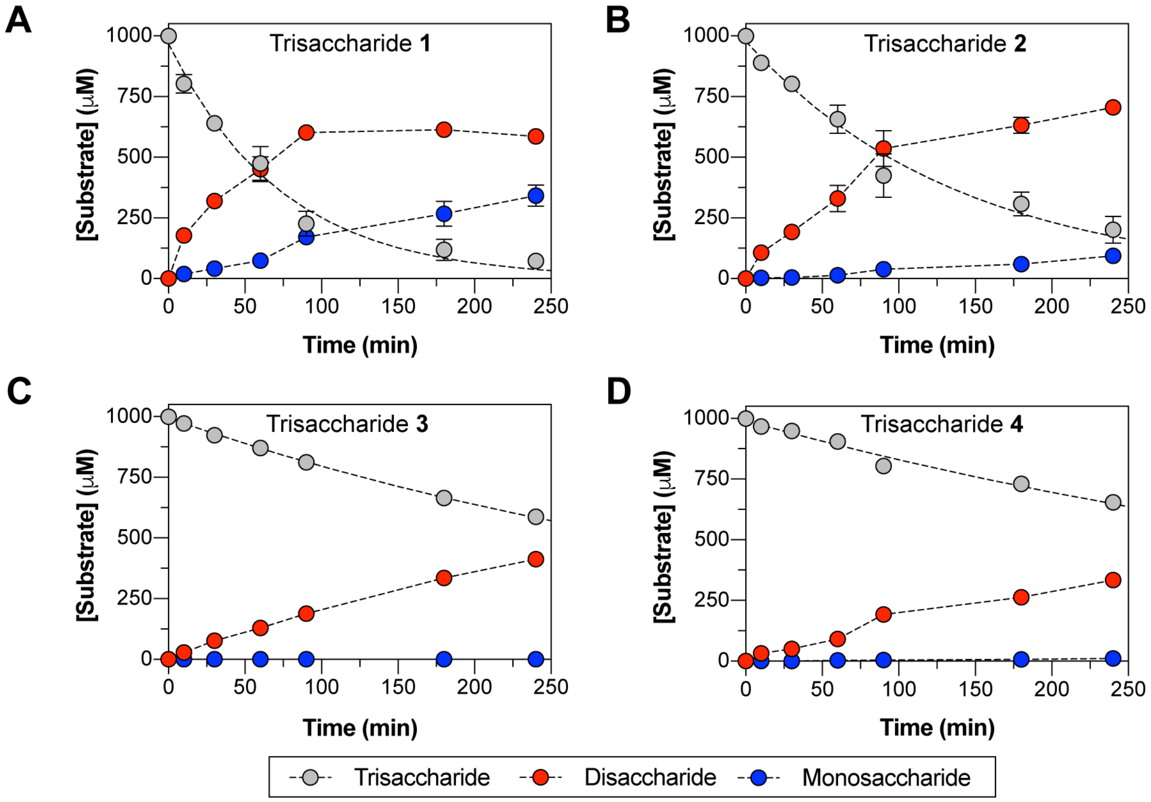

Figure S5. Hydrolysis of PNAG analogs 1–4 by DspB E248Q mutant. Reaction progress curves for hydrolysis reactions containing 11 μM DspB E248Q and 1000 μM of trisaccharide analog **1** (A), **2** (B), **3** (C) or **4** (D) measured as a function of time. Error bars represent the standard deviation from at least two replicate experiments. Lines were added to aid identification of the disappearance of the trisaccharide (grey) and appearance of reducing-end disaccharide (red) and reducing-end monosaccharide (blue) products.

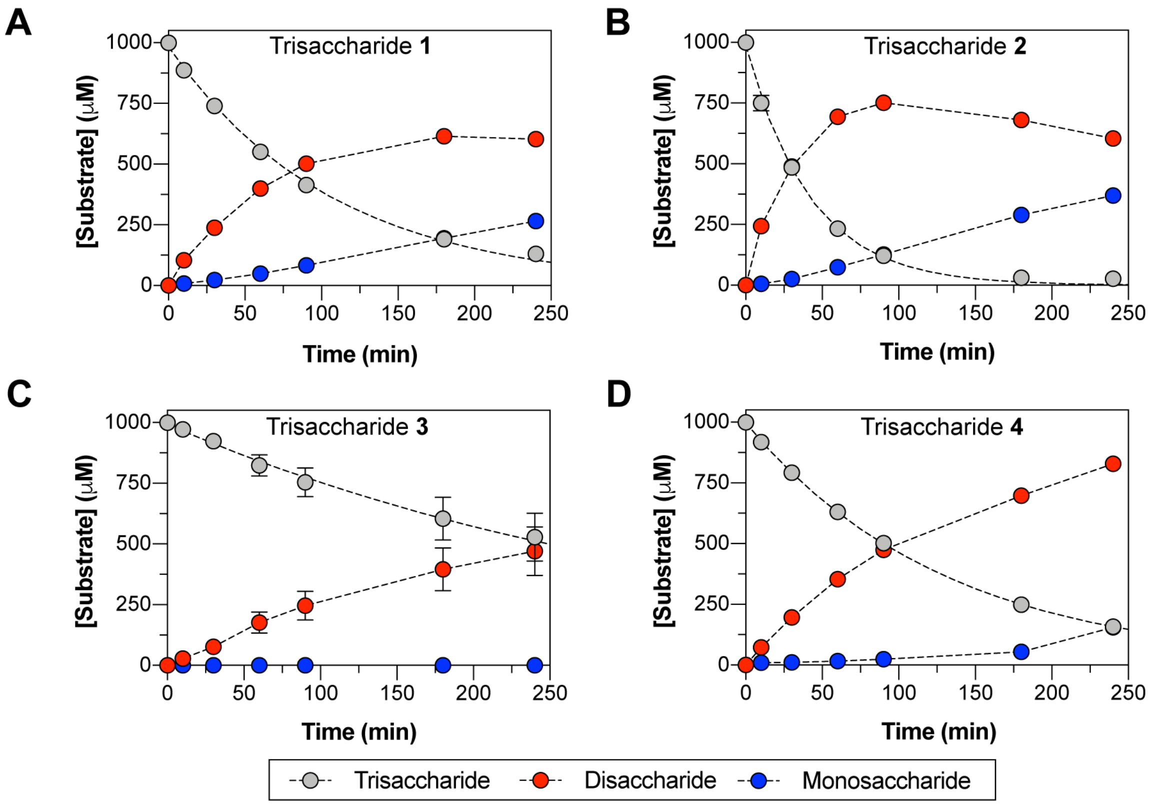

Figure S6. Hydrolysis of PNAG analogs 1–4 by DspB E248A mutant. Reaction progress curves for hydrolysis reactions containing 35 μM DspB E248A and 1000 μM of trisaccharide analog **1** (A), **2** (B), **3** (C) or **4** (D) measured as a function of time. Error bars represent the standard deviation from at least two replicate experiments. Lines were added to aid identification of the disappearance of the trisaccharide (grey) and appearance of reducing-end disaccharide (red) and reducing-end monosaccharide (blue) products.

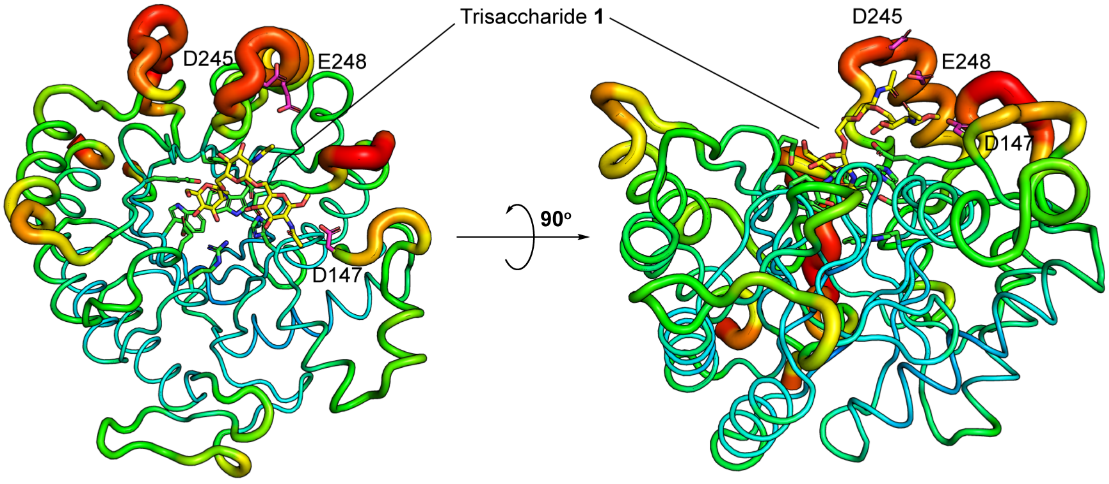

Figure S7. Analysis of thermal motion in DspB structure. The structure of DspB (PDB 1YHT) where the color and ribbon thickness denote the B-factors. Residues are colored according to B-factor on a scale ranging from cyan (B-factor = 3) to Red (B-factor = 54) with thicker ribbon denoting higher B-factor. The position of D147, D245, and E248 are shown as magenta sticks. The structure of docked trisaccharide **1** is shown as yellow sticks and conserved catalytic site residues are shown as green sticks.

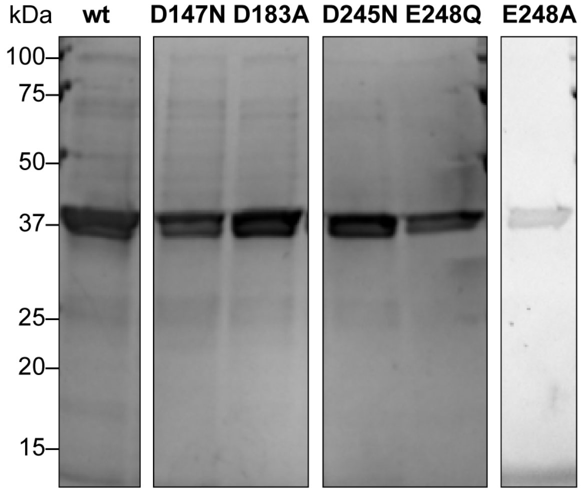

Figure S8. SDS-PAGE analysis of DspB wild type and mutant enzymes. All enzymes were prepared to ≥95% purity as seen by SDS-PAGE.

3. SPECTRAL DATA

***p*-Tolyl 3,4-di-*O*-acetyl-6-*O*-fluorenylmethyloxycarbonyl-2-deoxy-2-phthalimido-*β*-D-glucopyranosyl-(1→6)-3,4-di-*O*-acetyl-2-deoxy-2-phthalimido-*β*-D-glucopyranosyl-(1→6)-2,3,4-tri-*O*-acetyl-1-thio-*β*-D-glucopyranoside (8)**

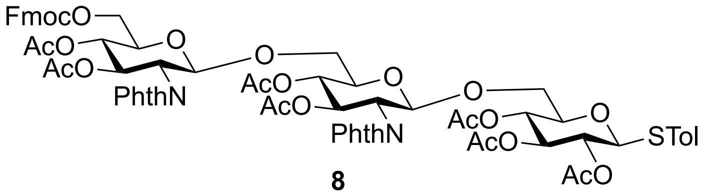

8

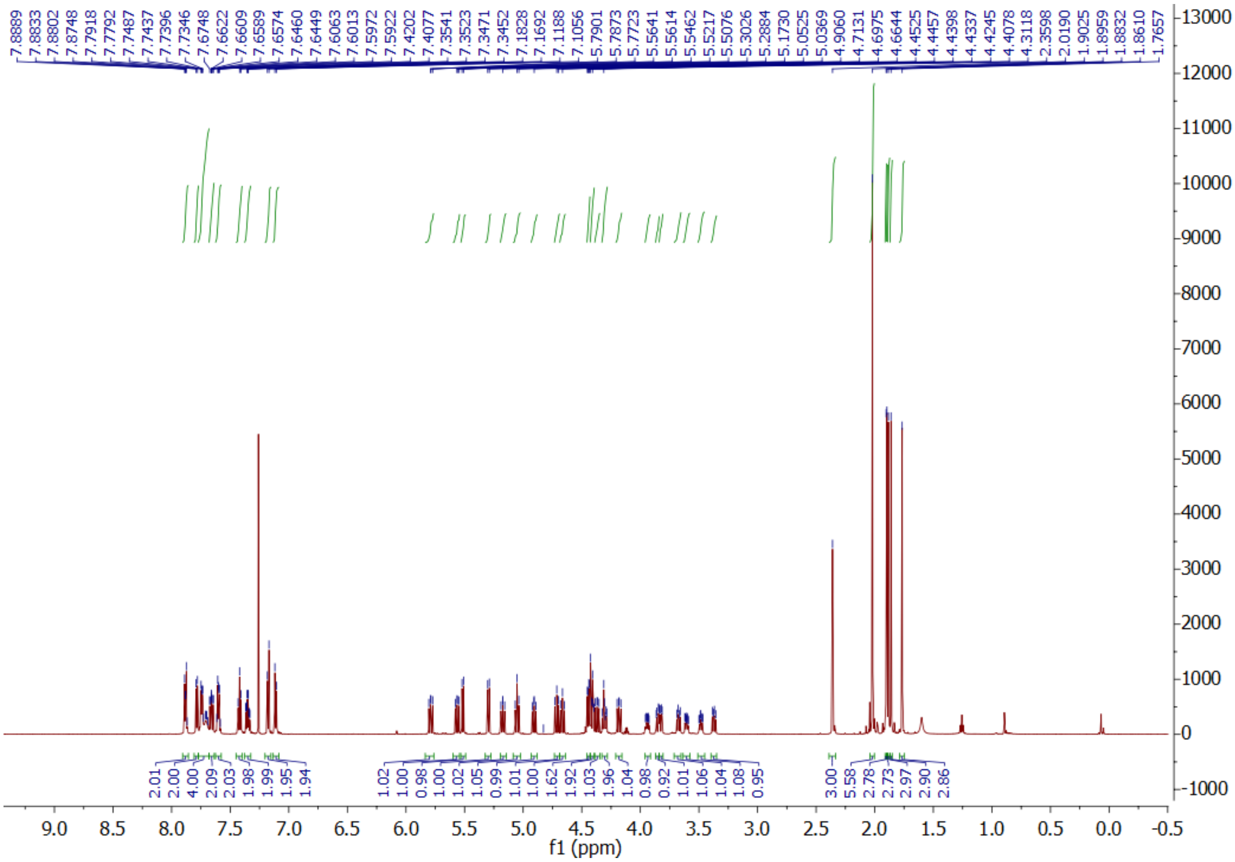

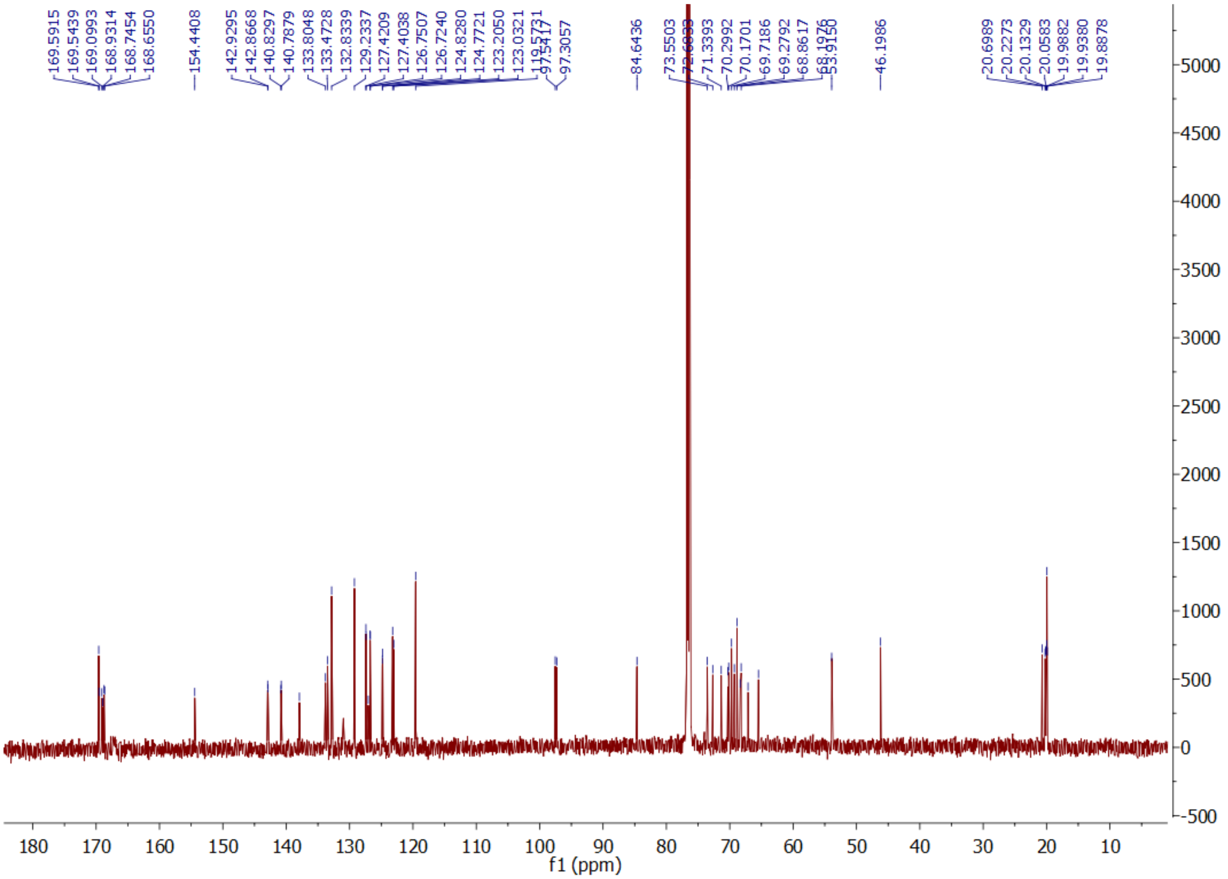

***p*-Tolyl 3,4,6-tri-*O*-acetyl-2-acetamido-2-deoxy-*β*-D-glucopyranosyl-(1→6)-3,4-di-*O*-acetyl-2-acetamido-2-deoxy-*β*-D-glucopyranosyl-(1→6)-2,3,4-tri-*O*-acetyl-1-thio-*β*-D-glucopyranoside (9)**

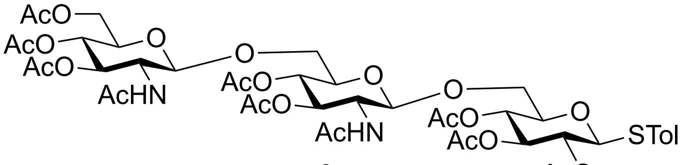

9

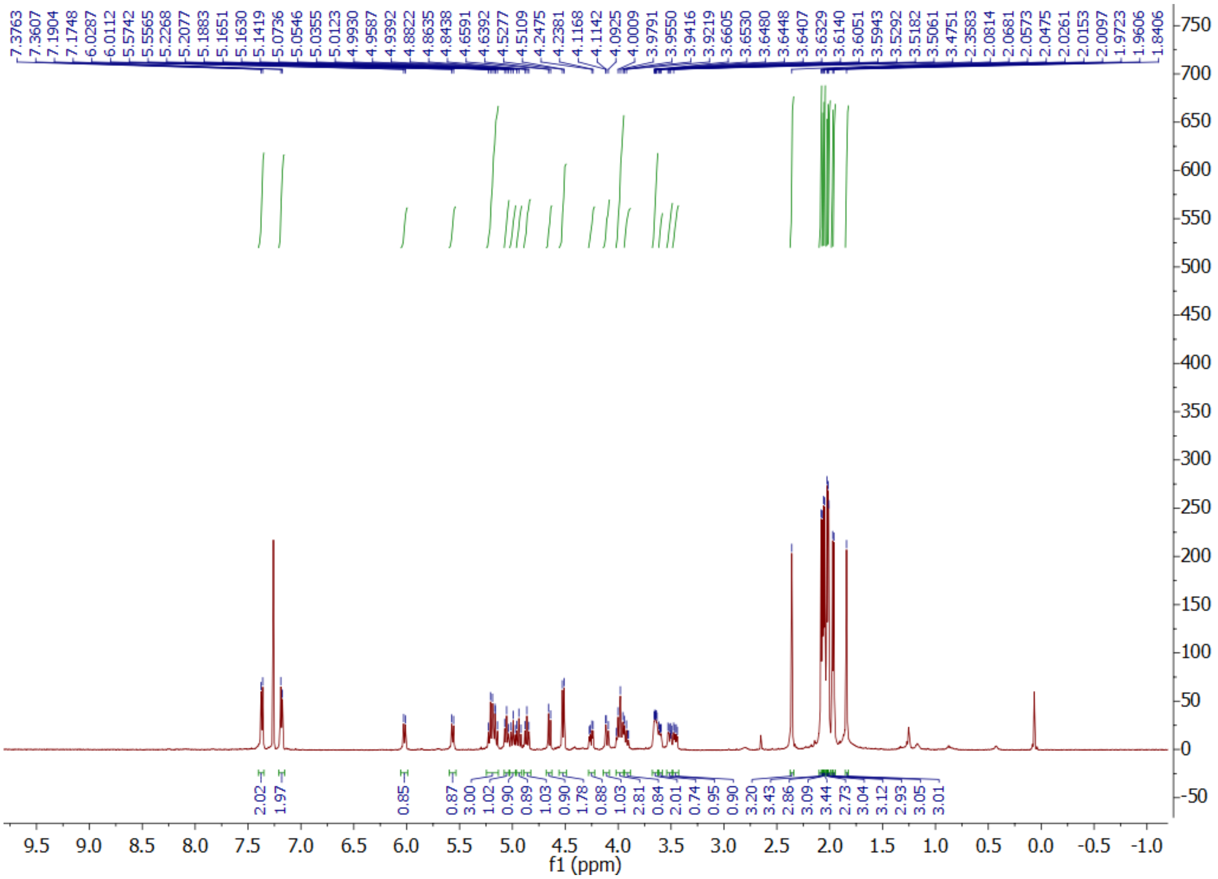

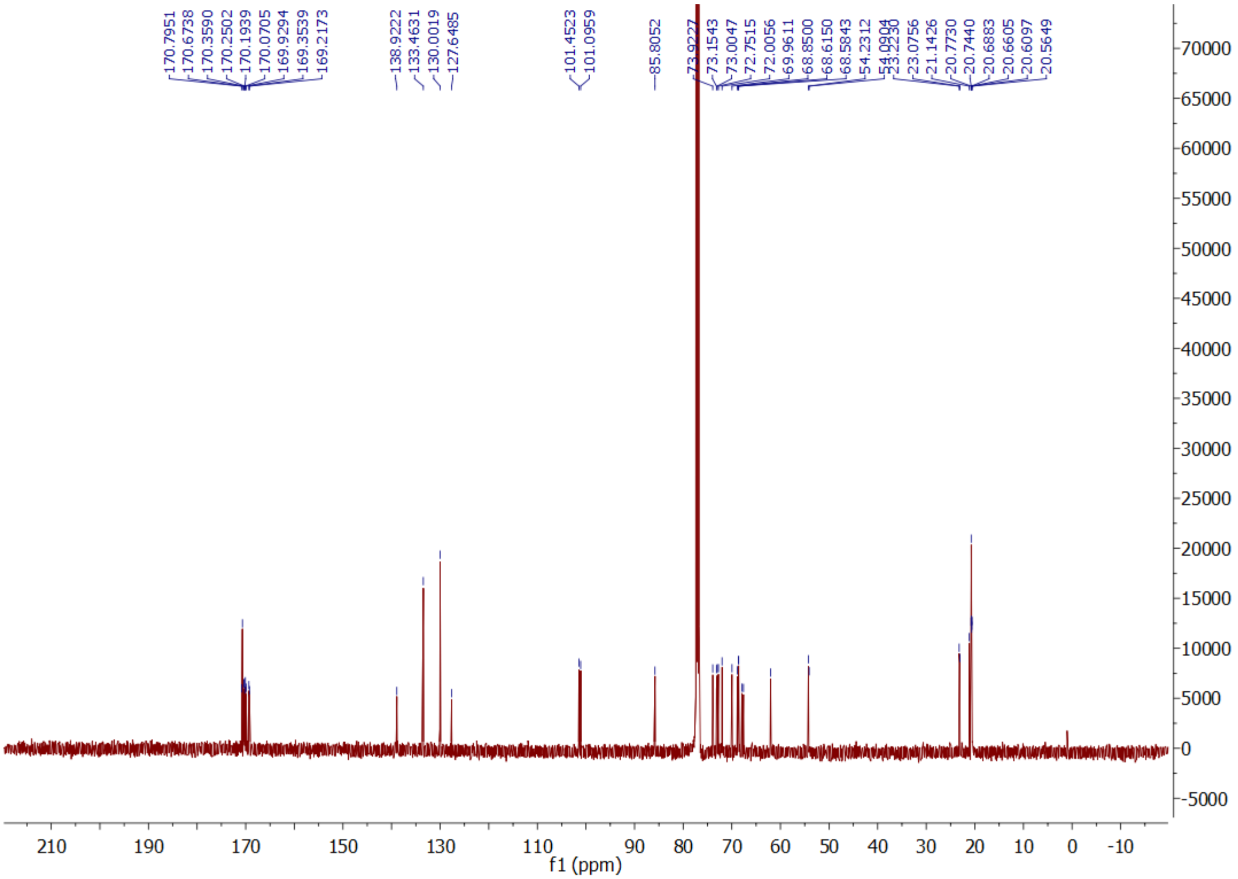
170.7951
170.6738
170.3590
170.2502
170.1939
170.0705
169.9294
169.3539
169.2173
138.9222
133.4631
130.0019
127.6485
101.4523
101.0959
85.8052
73.9227
73.1543
73.0047
72.7515
72.0056
69.9611
68.8500
68.6150
68.5843
54.2312
23.0756
21.1426
20.7730
20.7440
20.6883
20.6605
20.6097
20.5649
f1 (ppm)

p-Methylphenyl 2-acetamido-2-deoxy-β-D-glucopyranosyl-(1→6)-2-acetamido-2-deoxy-β-D-glucopyranosyl-(1→6)-1-thio-β-D-glucopyranoside (4)

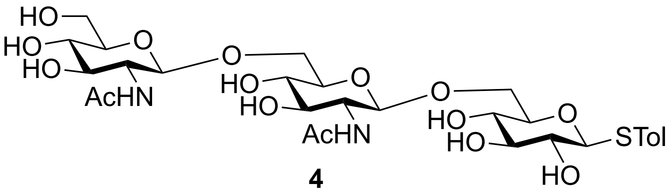

4

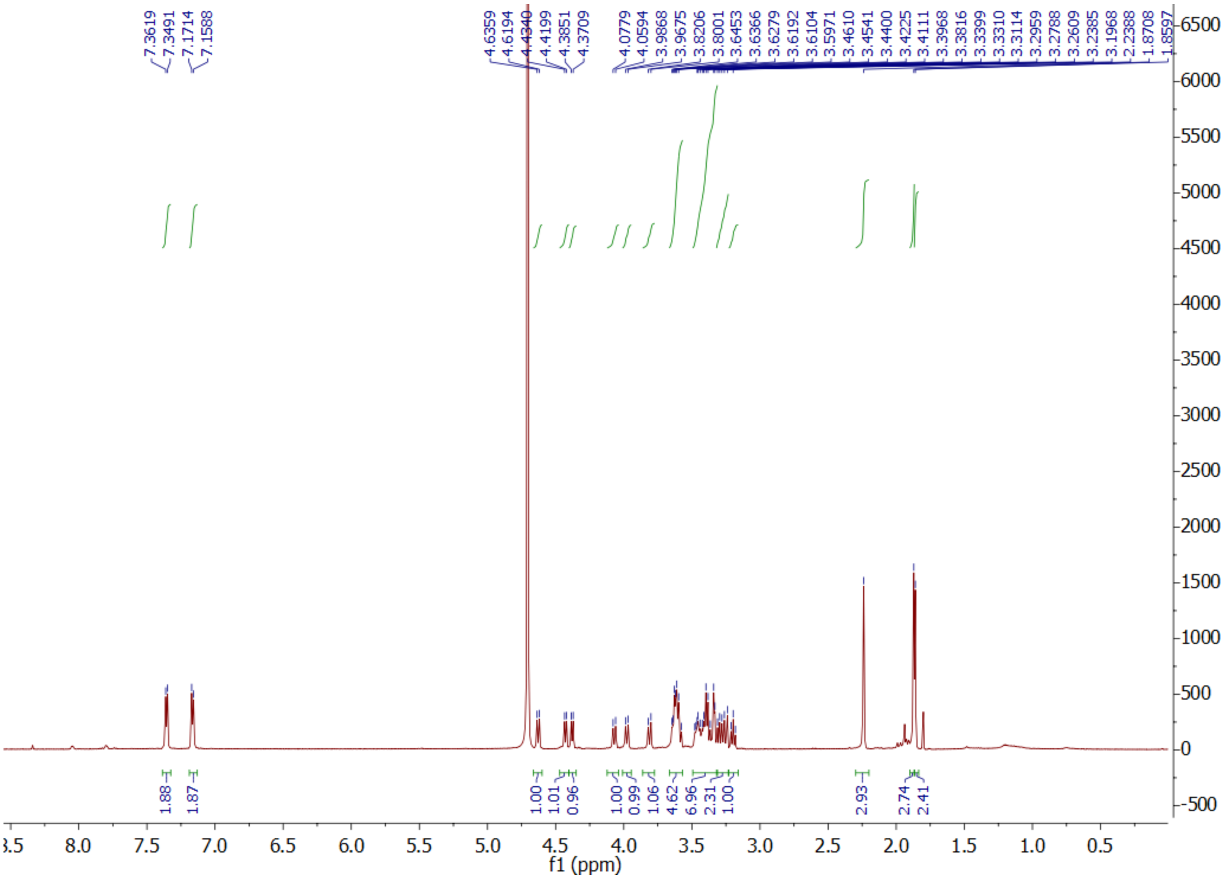

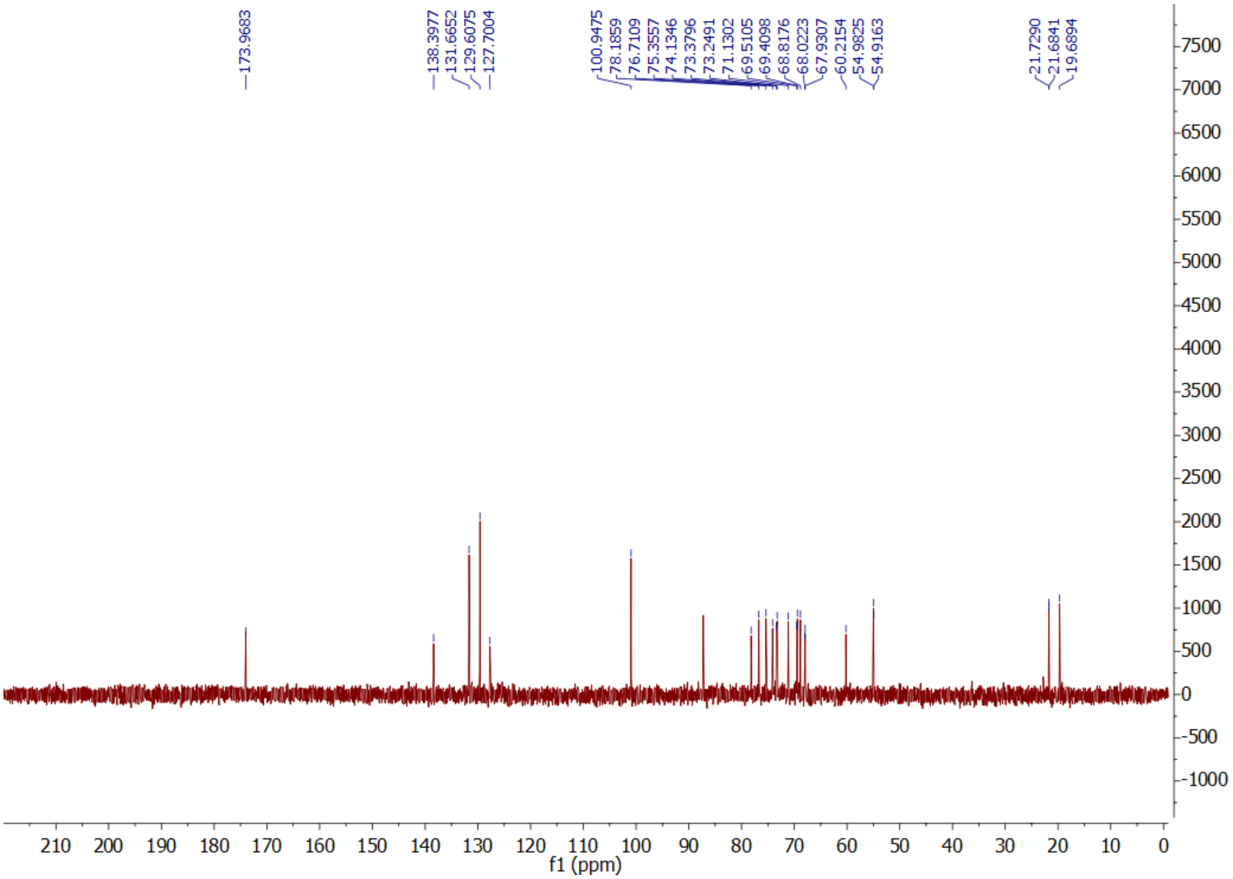

173.9683
138.3977
131.6652
129.6075
127.7004
100.9475
78.1859
76.7109
75.3557
74.1346
73.3796
73.2491
71.1302
69.5105
69.4098
68.8176
68.0223
67.9307
60.2154
54.9825
54.9163
21.7290
21.6841
19.6894
f1 (ppm)
